# Stabilization of a *p*-*u* Sensor Mounted on a Vehicle for Measuring the Acoustic Impedance of Road Surfaces

**DOI:** 10.3390/s20051239

**Published:** 2020-02-25

**Authors:** Francesco Bianco, Luca Fredianelli, Fabio Lo Castro, Paolo Gagliardi, Francesco Fidecaro, Gaetano Licitra

**Affiliations:** 1 iPOOL S.r.l., via Cocchi 7, 56121 Pisa, Italy; francesco.bianco@i-pool.it; 2 Physics Department, University of Pisa, Largo Bruno Pontecorvo 3, 56127 Pisa, Italy; fredianelli@df.unipi.it (L.F.); paolo.gagliardi@df.unipi.it (P.G.); francesco.fidecaro@unipi.it (F.F.); 3 CNR-INM Section of Acoustics and Sensors O.M. Corbino, via del Fosso del Cavaliere 100, 00133 Rome, Italy; fabio.locastro@cnr.it; 4 Environmental Protection Agency of Tuscany Region, via Vittorio Veneto 27, 56127 Pisa, Italy

**Keywords:** p-u sensor, p-p sensor, noise, Adrienne, stabilization, damping, acoustic impedance, road surfaces

## Abstract

The knowledge of the acoustic impedance of a material allows for the calculation of its acoustic absorption. Impedance can also be linked to structural and physical proprieties of materials. However, while the impedance of pavement samples in laboratory conditions can usually be measured with high accuracy using devices such as the impedance tube, complete in-situ evaluation results are less accurate than the laboratory results and is so time consuming that a full scale implementation of in-situ evaluations is practically impossible. Such a system could provide information on the homogeneity and the correct laying of an installation, which is proven to be directly linked to its acoustic emission properties. The present work studies the development of a measurement instrument which can be fastened through holding elements to a moving laboratory (i.e., a vehicle). This device overcomes the issues that afflict traditional in-situ measurements, such as the impossibility to perform a continuous spatial characterization of a given pavement in order to yield a direct evaluation of the surface’s quality. The instrumentation has been uncoupled from the vehicle’s frame with a system including a Proportional Integral Derivative (PID) controller, studied to maintain the system at a fixed distance from the ground and to reduce damping. The stabilization of this device and the measurement system itself are evaluated and compared to the traditional one.

## 1. Introduction

Transport infrastructures continue to be a source of noise, representing a serious issue in modern society. Among the sources that should be monitored according to European Environmental Noise Directive (END) [1], roads are the one that reach most citizens, and even if the noise produced is not the most disturbing, they contribute the most to the overall noise exposure. According to the 2017 END’s review [2], nearly 100 million European citizens are exposed to road traffic *Lden* higher than 55 dB(A) and among them 32 million are exposed to *Lden* higher than 65 dB(A). Many studies show that prolonged exposure to noise can induce cardiovascular disease [3,4], alterations in blood pressure [5,6], respiratory diseases [7], hypertension [8], learning impairment [9,10], annoyance [11,12], and sleep disturbance [13,14,15].

In order to avoid the aforementioned health issues, the END set the instruments in mandatory action plans for big infrastructures and urban agglomerations [16]. More detailed noise maps are mandatory for roads with traffic flows greater than 3 million vehicles per year and mitigation should be applied when limits’ exceedances emerge. In the last decades, economic efforts have been spent in mitigation actions for road traffic noise, with rigid barriers used as an optimal choice. However, it has been proven that this solution suffers from several problems, such as diffraction at the edges, reflection of sound energy in the opposite direction, and complaints of citizens due to the reduction of fields of view, natural light, and air flow [17].

Thus, researchers are recently looking for innovative and possibly “green” solutions, such as live and integrated monitoring systems [18,19], sustainable metamaterial absorbers [20,21], sonic crystals noise barriers made of recycled materials [22], electric cars, and car sharing [23].

The overall noise produced by a vehicle is given by the sum of propulsion, tire-pavement interaction, and aerodynamic contribution, depending on the vehicle speed. As electric vehicles become more widespread, the contribution of engines to overall noise is decreasing; however, it is only relevant at low speeds. Noise produced by tire–road interaction is the responsible for most of the noise emitted by roads and is a complex phenomenon resulting from the combination of aerodynamic and vibro-dynamic phenomena [24,25]. The former is related to compression of the air trapped within the tread of the rolling tire [26] (known as air pumping) and cause noise at frequencies higher than 1 kHz. In addition, pipe and Helmholtz resonances due to the coupling of a vibrating mass of air within the tread acting as a cavity contribute to the aerodynamic noise. Vibro-dynamic noise covers frequencies lower than 1 kHz and is due to tire vibrations caused by the impact of the tire against irregularities of the road surface and by non-linear effects, such as the stick-and-slip and stick-and-snap mechanisms [25].

The knowledge of noise generation mechanisms is vital for improving mitigation actions [27] applied to the two main actors in the phenomenon: Tires have been optimized [28], and mostly, low noise pavements have recently spread, some of which have even been developed with recycled materials [29,30]. Since tire/road noise depends on structural parameters of road surfaces [31], the European Union has recently prescribed acoustic tests of newly laid surfaces [32] by means of the Close Proximity Index (CPX) method [33] during the first three months from laying date. Therefore, knowing the acoustic performance of a pavement plays an extremely important role [34,35].

Even after the laying, monitoring a pavement over time is important to keep track of the ageing effect, which dramatically reduces the acoustic performance of surfaces. Traffic and weather cause voids in the surface to clog with detritus, increasing noise emitted even by 5 dB [36,37].

CPX measurements are generally performed to evaluate the acoustic emission properties of a pavement, while other measurement methods are needed for a wider evaluation of the acoustic properties. In order to assess the effects of noise on the receptors, for example, other factors are involved besides the emission, such as acoustic propagation, which in turn depends on the absorption coefficient of the road surface. The evaluation of the absorption coefficient is also an important parameter related to the state of the installation. For this reason, being able to measure the absorption yields a broader characterization of the pavement. In fact, the surface texture and volumetric of the friction course of a pavement are the main parameters influencing rolling noise, also related to absorption coefficient [35,36,37,38,39].

Different methods are generally used to evaluate the acoustic properties of a pavement, in order to derive information about their use in practical applications: From the CPX method [40], that also evaluates the emission properties, to the absorption measurement performed with an impedance tube using standing wave ratio (ISO 10534-1) [41] in-lab or on-site (ISO 13472-2) [42], and the extended surface method, usually called “Adrienne method” (ISO 13472-1) [43,44]. A recent study [45,46] showed that Adrienne method achieves good estimates for pavement’s characteristics, whereas the impedance tube scores the best accuracy in-lab, at the expense of time consumption, and the on-site Kundt tube can give an acceptable estimate of porosity.

Models of surface impedance of the materials currently present in the literature (e.g., Allard and Atalla [47]) are based on parameters related to the geometric properties of the interconnected porous structure and therefore to the effective structure of the medium. The sound absorption is often used as a proxy of the impedance, however, knowledge on the impedance represents a more complete information and allows a better characterization of surfaces from an acoustical point of view. Thus, in the last decade, several authors explored the possibility of measuring the surface impedance, defined as the ratio between the local pressure *p* and particle velocity *u*. This measurement is usually carried out by using a pressure-velocity (*p*-*u*) probe. The convenience of using a p-u probe is twofold: On one hand, it provides a local and simultaneous measure of both pressure and particle speed, while on the other hand it allows a direct measure of the impedance. A direct measurement of velocity is not possible with other known methods, such as the *p*-*p* sensors (pressure – pressure) reported below but can be achieved indirectly. In this way, the two quantities are related to the same incident and reflected field, with consequently simpler and error-free calculations. The Adrienne method, for example, requires evaluating a direct field that requires subsequent subtraction from the measured one, but is clearly not stable over time. Another important advantage of using the p-u probe to measure the impedance/absorption coefficient is the broadband "figure of eight" directivity of the particle velocity sensor, which significantly restricts background noise and allows the sensor to be used in-situ.

Most of the studies used the probe in laboratory conditions [48,49], while, to the best of the authors’ knowledge, only few approaches have tried in-situ road surface impedance measurement [50,51,52]. Nevertheless, Tijs and de Bree’s concept [53] can be considered a precursor of the present work, as it uses a small source attached to the bumper and close to the road surface and a p-u probe, operating in near field conditions and using a spherical wave.

The methodology reported in the paper uses a p-u sensor that measures the sound intensity by means of a pressure transducer combined with a particle velocity transducer, instead of the more common *p*-*p* sensor combining two pressure microphones and then applying a finite-difference approximation to the pressure gradient. Random errors can be overcome by repeating the measurement, while bias errors can occur for multiple causes, such as phase mismatch between the two measurement channels, errors in the scanning procedure, errors due to airflow, influence of the pressure equalization vents of the microphones, non-stationary external sources, reflections from the operator as they move around the source, influence of the environment on the sound power output of the source, and the absorption of the source itself [54]. These reasons contribute to the choice of the *p*-*u* probe over a *p*-*p* one. Furthermore, background noise from sources outside the measurement surface can increase phase mismatch in *p*-*p* sensors, but not in p-u ones [55]. On the contrary, strongly reactive sound fields can increase *p*-*u* phase mismatch and have no influence with the *p*-*p* ones. Another advantage of p-u probes over *p*-*p* ones is their reduced size; however, *p*-*u* sensors are not easily calibrated.

In the framework of the NEREiDE LIFE project, a new measurement system based on a pressure-velocity (*p*-*u*) probe has been implemented on a mobile laboratory. The present paper describes an instrument mounted on a mobile laboratory able to measure the absorption coefficient of a pavement in a continuous way. A mobile laboratory allows in-situ measurements of long road sections for a more precise assessment of the quality of asphalt pavements, which can vary along the installation [56]. It also allows to make measurements in safe conditions and without necessarily closing the road to traffic.

After addressing the choice of the sensor used, this work tries to solve the primary issue regarding these measurements, which is the stabilization of the instrumentation respect to the road surface. The new approach has been derived from the Adrienne method and can measure the acoustic absorption in a contactless way, thus resulting in a wider frequency bandwidth and a larger studied portion of the road surface. A proper device, consisting of an actuator driven by a laser distance sensor, has been developed for stabilizing and damping the measuring system. This task is extremely important for maintaining the stability and correctness of the impedance evaluation and reducing the measuring errors. Its effects have been simulated and measured in order to validate its functioning.

## 2. Life NEREIDE Project

The present work is part of the LIFE15 ENV/IT/000268 NEREiDE project, which aims improve porous asphalt pavements and low noise surfaces made of recycled asphalts and tires. A warm mixture was produced at a high temperature giving birth to pavements that improve:

- safety in urban areas by better draining;
- the reduction of waste materials and virgin materials;
- acoustical performances and a significant reduction of noise emitted;
- asphalt laying procedure, thus reducing air pollution emissions.

Crumb rubber from scrap tires were used in order to improve the road surfaces from elastics, soundproofing and toughening characteristics points of view, while recycled asphalts were used to reduce virgin aggregates and bitumen. The project characterizes raw materials searching for the best that fit the manufacturing processes of crumb rubber in order to modify the vulcanized recycled rubber and to make it suitable for incorporation in the bitumen. The last was the binder and matrix of the new composite material, with structural and waterproofing functions that should avoid the leaching of chemicals. Laboratory tests had already selected the correct composition, grading curve and the structural characteristics, the percentage of crumb rubber, and the methods to realize them.

The new pavements had been laid in two urban areas in Tuscany and one of the major tasks of the project was the evaluation of their effectiveness. Different approaches are currently used to test their effectiveness; from surface characteristics, to acoustical properties, and surveys submitted to the exposed population. The effectiveness was evaluated through a comparison of the surface acoustical properties prior and after the laying, but also by comparing the results with measurements over standard porous asphalt. This led to another objective, which is not less important than the main one: To improve the reliability of the results by suggesting a new evaluation technique for the performances of new pavements. The method should work on-site using techniques also applicable in urban context. Then, the effectiveness of new asphalts was monitored, also considering the subjective response to noise ante and post-opera. Roadside noise measurements were used to validate outputs from a noise model in order to assign noise levels to nearby residents who were also interviewed with questionnaires that compared their response [57].

Both Close Proximity Index and statistical pass-by (SPB) methods [58] were used to measure the noise produced by tires rolling on new and reference pavements. Unfortunately, standard SPB is applicable under environmental conditions that are almost never realized in the urban context. Therefore, an urban statistical pass by method was developed adding a monitoring station placed roadside at a known distance, in addition to traffic counters. In this way, the SPB has been modified to be used in urban areas by reducing the confounding factors. The method for deriving pass by values in urban contexts can define the index of vehicles noisiness on the specific pavement and place, thus allowing a comparison of efficiency of new pavements in terms of noise at roadside.

The project also aimed at the development of a new on-site acoustical absorption measuring system built on a mobile laboratory. For this purpose, a car was equipped with several instruments in order to perform moving and static measures. A preliminary data analysis was set up and described in the present paper. The system was used to monitor acoustic properties of new surfaces realized on experimental sites and was able to give real time results and a fine spatial acoustic characterization of the road surface. The results were linked to the microscopic and macroscopic properties of the asphalt, with parameters such as porosity and tortuosity, but also to macroscopic discontinuities or structural problems. They also provide an evaluation of the current state of a whole pavement stretch, thus giving useful information on the homogeneity of results along the whole installation. This allows for the evaluation of the goodness of the mixture and its laying. In the rest of the paper, a description of the measurement’s methodology will be carried out, focusing on the difficulties regarding the stabilization of the system over a moving vehicle.

## 3. Methodology

Measuring the absorption coefficient with a moving vehicle is best achieved using a contactless method. The system used in this work is the sum of the following parts, each separately tested and calibrated both in controlled conditions and on the actual mobile laboratory:

(1) p-u probe, also known as intensity probes: A sensor measuring the local value of acoustic pressure (*p*) and particle velocity (*u*). With these two parameters, the probe can easily evaluate the acoustic impedance; 

(2) inertial damper: An electro mechanic actuator driven by a laser distance measure between the car and the pavement surface. This tool is needed to maintain constant the distance between the probe and the asphalt and to reduce vibrations from the moving vehicle;

(3) acoustic emitter: The sound emitted by the source is measured by the probe after being reflected by the pavement;

(4) A pc and an acquisition board acting as instrumentation control system.

**Figure 1:**
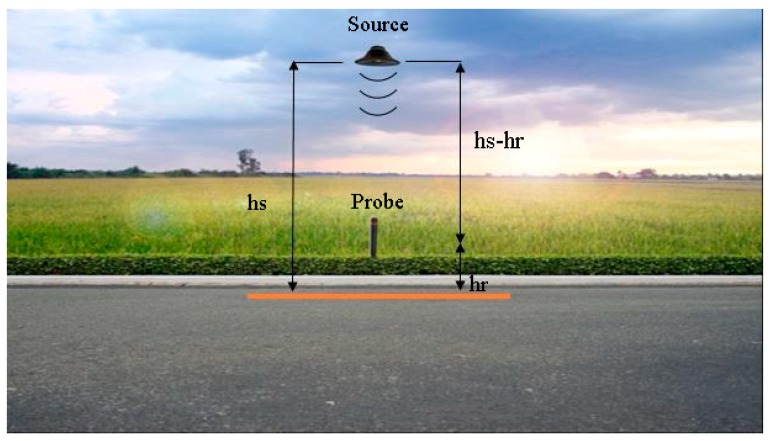
Set-up of the measurement system.

The p-u probe can simultaneously and directly detect local pressure and particle velocity. It consists of a miniaturized pre-amplified microphone for pressure measurements and hot wires for the particle velocity evaluation [59]. This instrument is sensitive to specific velocity direction. As reported in Figure 1, the velocity sensor measures the perpendicular component respect to the pavement. An older method for measuring the particle’s velocity consisted of using two matched microphones and calculating the pressure difference, but problems in low pressure variation occurred when considering the microphones dimension and the spacing between them. The introduction of a technique based on a hot wire overcome this issue and the particle velocity could then be evaluated with a wire whose electric resistance changes with the temperature, and hence with the speed of the flow of the air that flows over the wire. The velocity *u* was then evaluated with Equation (1) [60].

(1) u=(E2−ab)1n

where *E* is the voltage difference at the two ends of the wire resistance, *a*, *b,* and *n* are three constants evaluated during calibration. The voltage across the resistance is given by Equation (2) [61].

(2) $$E=(R_{0}+\Delta R)i$$

where *R_o_* is the value of the resistance at the temperature *T_o_* and the resistance variation ΔR is given by Equation (3), where α is thermal resistance coefficient and *T* the temperature of the hot wire.

(3) $$\Delta R=R_{0}\alpha \left(T-T_{0}\right)$$

The model used in this work considers a monopole source placed over the ground at *h_s_* and a receiver with height *h_r_* from the ground, as shown in Figure 1. The receiver acquires both *p* and *u* [62]. An innovative stabilization system is used to reduce the variation of the height of the source and the receiver, and it will be described in the next chapter. 

The acoustic impedance was calculated with Equation (4) starting from the ratio of the complex pressure to complex velocity amplitudes measured by the probe.

(4) $$Z_{r}\left(r,\omega \right)=\frac{p_{r}\left(r,\omega \right)}{u_{r}\left(r,\omega \right)}$$

The pressure and velocity values were calculated with Equations (5) considering a spherical wave of radius *r*:

(5) $$p\left(r,t\right)=\frac{A}{r}e^{-kri}e^{i\omega t}\;u\left(r,t\right)=\frac{A}{\rho cr}\left(1-\frac{i}{kr}\right)e^{-kri}e^{i\omega t}$$

*A* is the wave amplitude, ρ is the air density, *c* is the speed of sound, ω=2πf is the angular frequency, $k=\frac{2\pi }{\lambda }=\frac{2\pi f}{c}$ is the acoustic wave number, λ is the wavelength.

The absorption coefficient *α* is defined as α=1−Γ, where Γ is the reflection coefficient defined in Equation 6 as the ratio of the reflected power *P_r_* and incident power *P_i_*.

(6) $$\Gamma =\frac{P_{r}}{P_{i}}≅\frac{{\left|p_{r}\right|}^{2}}{{\left|p_{i}\right|}^{2}}={\left|R\right|}^{2}$$

*R* is the sound pressure reflection factor. *p* is the sum of the direct and reflected pressure wave. Similarly, *u* is the sum of direct and reflected velocity wave. However, since they are vectors, the negative sign must be taken into account. The impedance can then be reported in Equation (7).

(7) $$Z_{r}\left(r,t\right)=\;\frac{p(h_{s}-h_{r},t)+p(h_{s}+h_{r},t)}{u(h_{s}-h_{r},t)-u(h_{s}+h_{r},t)}=\;=\frac{\frac{A}{d_{1}}e^{-kd_{1}i}e^{i\omega t}+R\frac{A}{d_{2}}e^{-kd_{2}i}e^{i\omega t}}{\frac{A}{\rho cd_{1}}\left(1-\frac{i}{kd_{1}}\right)e^{-kd_{1}i}e^{i\omega t}-R\frac{A}{\rho cd_{2}}\left(1-\frac{i}{kd_{2}}\right)e^{-kd_{2}i}e^{i\omega t}}\;=\;=\rho c\frac{\frac{1}{d_{1}}e^{-kd_{1}i}+R\frac{1}{d_{2}}e^{-kd_{2}i}}{\frac{1}{d_{1}}\left(1-\frac{i}{kd_{1}}\right)e^{-kd_{1}i}-R\frac{1}{d_{2}}\left(1-\frac{i}{kd_{2}}\right)e^{-kd_{2}i}}$$

where for simplicity $d_{1}=\left(h_{s}-h_{r}\right)$ and $d_{2}=\left(h_{s}+h_{r}\right).$ According to Equation (7), the explicit time dependency disappears and $Z_{r}\left(r,t\right)$ becomes in $Z_{r}\left(r\right)$ in the following. From the previous equation, *R* can be obtained Equations (8) and (9).

(8) $$R\left(r\right)=-\frac{\left(h_{s}+h_{r}\right)\left(\frac{Z_{r}\left[k\left(h_{r}-h_{s}\right)+i\right]}{\rho ck\left(h_{r}-h_{s}\right)}-1\right)}{\left(h_{r}-h_{s}\right)\left(\frac{Z_{r}\left[k\left(h_{s}+h_{r}\right)-i\right]}{\rho ck\left(h_{s}+h_{r}\right)}+1\right)}e^{-2kh_{r}i}$$

(9) $${\left|R\right|}^{2}={\left[\left(\frac{h_{s}+h_{r}}{h_{s}-h_{r}}\right)\left|\frac{\frac{Z_{r}\left[-k\left(h_{r}-h_{s}\right)-i\right]}{\rho ck\left(h_{r}-h_{s}\right)}+1}{\frac{Z_{r}\left[k\left(h_{r}+h_{s}\right)-i\right]}{\rho ck\left(h_{r}+h_{s}\right)}+1}\right|\right]}^{2}$$

In order to know which parameter mostly affects the absorption coefficient, according to the theory of propagation of uncertainty, the propagation error for a multivariable function is derived for each component from the partial derivative of the function itself. Thus, the sensitivity of *α* respect to $h_{s}$ or $h_{r}$ can be calculated with Equations (10).

(10) $$\frac{\partial \alpha }{\partial h_{r}}=\frac{\partial {\left|R\right|}^{2}}{\partial h_{r}}\;\frac{\partial \alpha }{\partial h_{s}}=\frac{\partial {\left|R\right|}^{2}}{\partial h_{s}}$$

## 4. Results

### 4.1. Instrumentation Height

Since it derives from the Adrienne method, the new method is dependent on the height from the plane. Considering that the height of the vehicle from the road surface changes due to its acceleration and to superficial irregularities of the surface, the very first part of the analysis are in-lab simulations aimed to optimize receiver’s position from the ground, which is the one leading to the smallest error in the calculation of the absorption coefficient. The results are compared with the Adrienne ones.

In these calculations, the ideal source and receiver were used and the variation of receiver to source height brought by the running vehicle were considered.

The sensitivity of *α* respect to the variation of $h_{s}$ or $h_{r}$ were studied in the Adrienne method with Equations (11).

(11) $$\frac{\partial \alpha }{\partial h_{r}}=\frac{\partial {\left|R\right|}^{2}}{\partial h_{r}}=-\frac{4h_{s}\left(h_{s}-h_{r}\right)}{{\left(h_{s}+h_{r}\right)}^{3}}{\left|\frac{P_{r}\left(f\right)}{P_{i}\left(f\right)}\right|}^{2}\;,\;\frac{\partial \alpha }{\partial h_{s}}=\frac{\partial {\left|R\right|}^{2}}{\partial h_{s}}=\frac{4h_{r}\left(h_{s}-h_{r}\right)}{{\left(h_{s}+h_{r}\right)}^{3}}{\left|\frac{P_{r}\left(f\right)}{P_{i}\left(f\right)}\right|}^{2}$$

From Equations (11), is possible to derive Equations (12), which shows that the ratio of the sensitivities of the absorption coefficients is equal to the ratio of $h_{s}$ and $h_{r}$. Therefore, the sensitivity to variation of height of receiver is higher in module than that respect to the variation of the height of source.

(12) $$\frac{\frac{\partial \alpha }{\partial h_{r}}}{\frac{\partial \alpha }{\partial h_{s}}}=-\frac{h_{s}}{h_{r}}$$

In the new method with a p-u probe as receiver, the sensitivities ratio is different and changes with frequency, pavement impedance, receiver, and source height. The sensitiveness of the absorption coefficient values obtained using the Adrienne method is far greater than the new method proposed. The absorption coefficient sensitivity to the variation of the height of the probe also results higher in the Adrienne method than in the new one.

The height of the probe corresponding to the lowest error is at 0.16 m from the pavement for the new method, while for Adrienne the height of the microphone is 0.25 m, as suggested by the standard. However, the error for the Adrienne method is higher than the new method around ±0.05 to the minimum of both curves.

The main features of Adrienne and the new method are summarized in Table 1. The new method offers a wider frequency band width and is less sensitive to variations of the receiver’s height, which makes it more appropriate for a running vehicle.

### 4.2. Damping System

**Figure 2:**
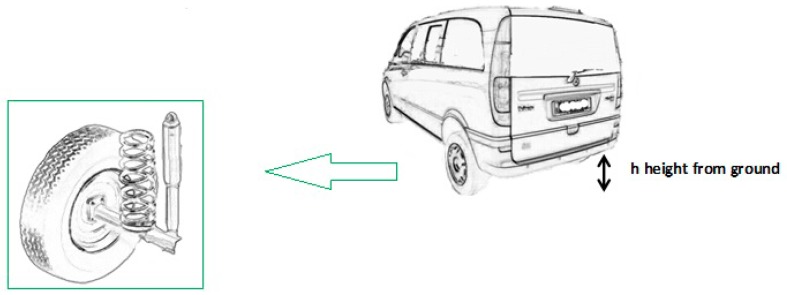
Example of vehicle where is installed the absorption measurement system, with a focus on the shock absorber.

**Figure 3:**
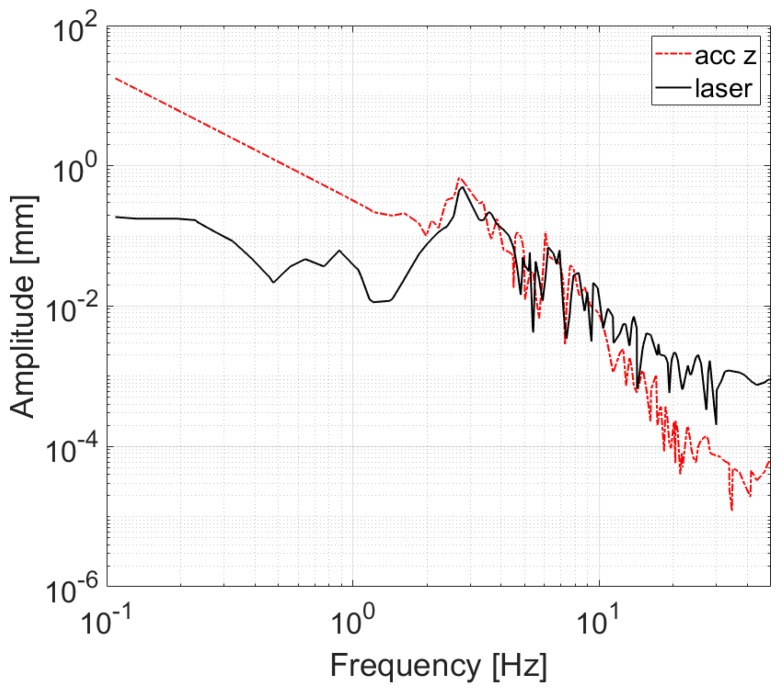
Example of vehicle vertical displacement spectrum while moving.

The mobile laboratory moves while acquiring data, therefore the height of the measurement instrument from the road pavement changes, as stated previously. The absorption measurement system was fastened to the frame of the vehicle and hence it suffered the same oscillations, shown in Figure 2. Figure 3 shows the vertical displacement of the rear of the vehicle, measured using an accelerometer and a laser distance meter. The accelerometer displacement was obtained through a double integration, which increased low frequency noise. On the contrary, the laser worked well over the whole band, but above a certain frequency the measurement was mainly influenced by the road texture (around 20 Hz). Red and blue curves in Figure 3 are similar within the range 2–20 Hz. Therefore, the spectrum of the searched displacement corresponds to the measurement performed with the laser up to that maximum identified frequency, while the following signal is attributable to the influence of road texture.

In order to reduce the influence of height variations on the measurement, the measurement instrumentation must be uncoupled from the vehicle frame, using a system capable of keeping a constant distance *h* from the pavement. The characteristics of this active damping system are hereby described.

**Figure 4:**
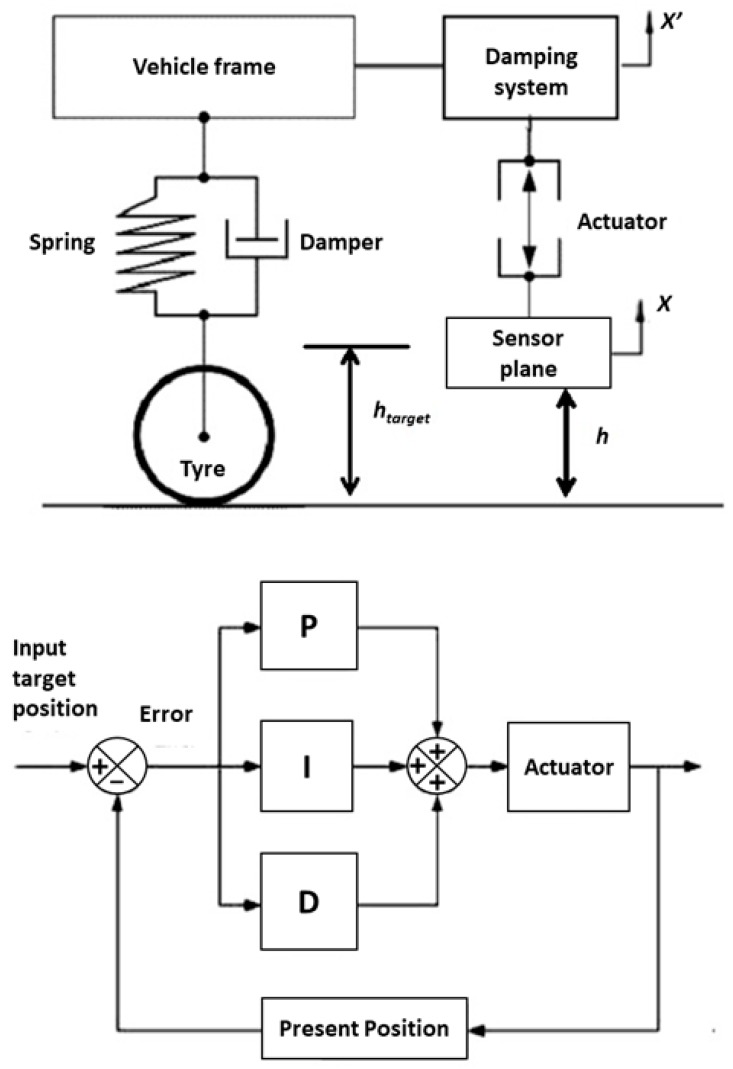
Damping system model and Proportional Integral Derivative (PID) controller of the height of the absorption measurement system.

As shown in Figure 4, an active controller on the distance *h* has been developed using a Proportional Integral Derivative (PID) controller with the following characteristics:

1. Maximum allowable displacement x_max_: 100 mm;
2. Damping system with 1 degree of freedom placed along vertical axis;
3. Typical vehicle frame displacement (x’): ±20 mm;
4. Displacement stabilization range (error = h_target_ -h): 2 mm ÷ 5 mm;
5. Working frequency: 0 Hz ÷ 30 Hz;
6. Actuator load: 3 kg payload (loudspeaker, sensors, windscreen, laser distance sensor) plus damping system frame.

The Proportional Integral Derivative (PID) controller acts on the error *e* equal to the difference of the target position $h_{target}$ and the current position *h* of the measurement system with respect to the pavement. The target position $h_{target}$ is defined as the almost fixed height of the system. Furthermore, it takes into account the error *e* itself through a proportional relation, its integral *I* and its derivative *D*. The error can be expressed with Equation (13).

(13) $$e=h_{target}-h$$

The function *f* used to drive the actuator in order to stabilize the measurement system is defined in Equation (14).

(14) $$f\left(e\right)=k_{p}e\left(t\right)+k_{I}\int_{0}^{t}e\left(t\right)dt+k_{D}\frac{\partial e\left(t\right)}{\partial t}$$

where $k_{p}$*,* $k_{I},k_{D}$ are constants to be calculated in accordance to the actuator system response and the acceptance error. In the present work, the accepted error was considered equal or less than 5 mm, corresponding to an absorption error due to the vehicle oscillation of around 0.01.

**Figure 5:**
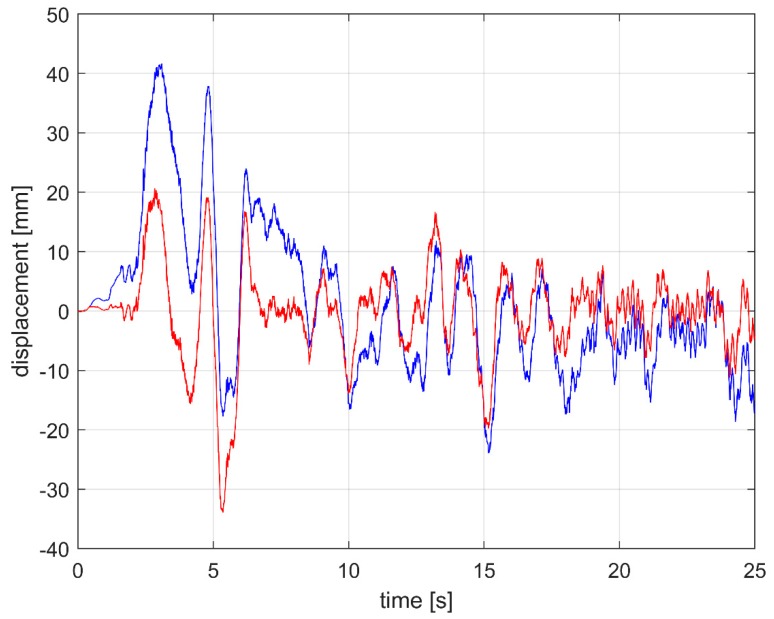
Displacement simulation of the sensor plate stabilized by a PID controller (red); acquired vehicle frame vibration used as input of the PID controller (blue).

A simulation of the process is reported in Figure 5. The control was made through the PID controller which acts on a linear slide actuator that keeps the sensor at a fixed height *h* from the pavement. The actuator linear speed used in the simulation is 20 mm/s, while the road profile is taken from the on-field measurement, described below.

A field test was carried out on a stretch of pavement in good condition, running on the same stretch with the stabilizer device off and on. The measurements were repeated several times at a speed of 30 km/h. Although it is not easy to repeat the exact condition of each run, the results showed good repeatability. Measurements with or without stabilizer are not contemporary because only one laser displacement sensor was available for measurements. Consequently, the two measurements do not correspond to the same exact profiles; however, it can be argued that the two profiles share the same mean properties since they derive from measurements of the same road surface.

**Figure 6:**
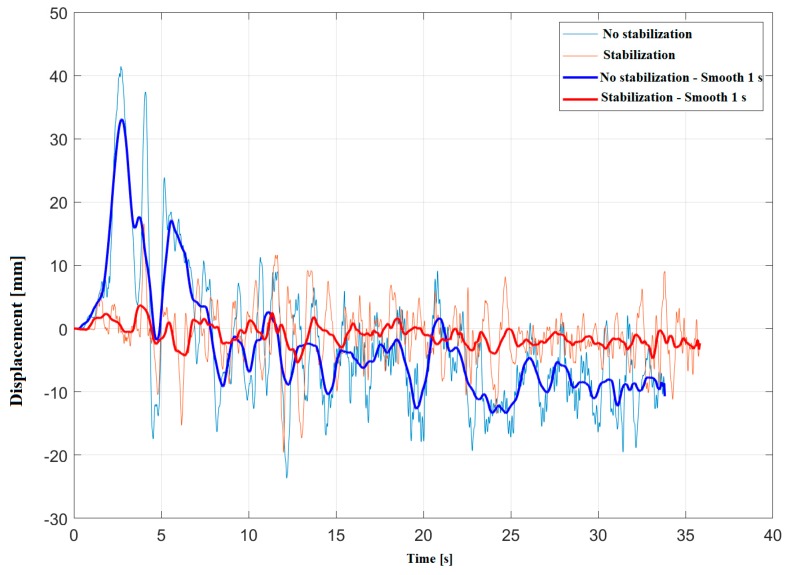
Displacement measured in-situ with stabilization (red) and without stabilization (blue), with smoothing (bold lines) and without smoothing (thin lines).

Figure 6 shows the displacements measured. The bold lines represent a 1-second smoothing for improving the visibility of the average stabilization effect. The maximum excursion using the stabilizer is approximately 0.021 m at 30 km/h. The order of magnitude of the average displacement from the expected equilibrium position in standard operating conditions are reported in Table 2.

In order to evaluate how using the stabilizer affects the absorption measurement, an idealized case where the only effective variable was the position of the sensor/speaker group relative to the surface investigated was taken into account. Thus, considering a perfectly homogeneous surface, which has the same spectral trend for the reflection coefficient at each point. Furthermore, a high directionality of the sensor is assumed, so that the area actually investigated can be considered constant.

The subsequent calculations were carried out considering a hypothetical pavement with absorption from almost zero to almost one along the whole frequency range, a vehicle in motion at 30 km/h and the displacement trends with respect to an equilibrium position of 0.16 m from the pavement, as in Figure 5, both for the stabilized and non-stabilized system.

In order to simplify the application of Equation (7), it was assumed that the system remains stable, i.e., at a constant distance from the pavement, within the single measurement made by the laser distance meter. Since the instrument works at a frequency of 64 Hz, the measurements were carried out every 15.6 ms, enough time to resolve rather low signal frequencies. For example, up to about five cycles can be measured at 315 Hz.

A measurement of the p and v signals was simulated in successive windows of 15.6 ms length, calculating for each the signal Z_r,i_ as the ratio of the respective spectra (i is the sections index). In order to do so, Equation (7) was used by imposing R, which was identical for each repetition, and the values of d_1,i_ and d2_,i_ related to the section. Using the calculated impedances but imposing a fixed distance of 0.16 m, the reflection coefficients R_i_, representing an estimate of the error made in the absence of stabilization or with an imperfect one, were obtained. Calculating the relative error for each section becomes simple, since the actual value that R must assume is known.

**Figure 7:**
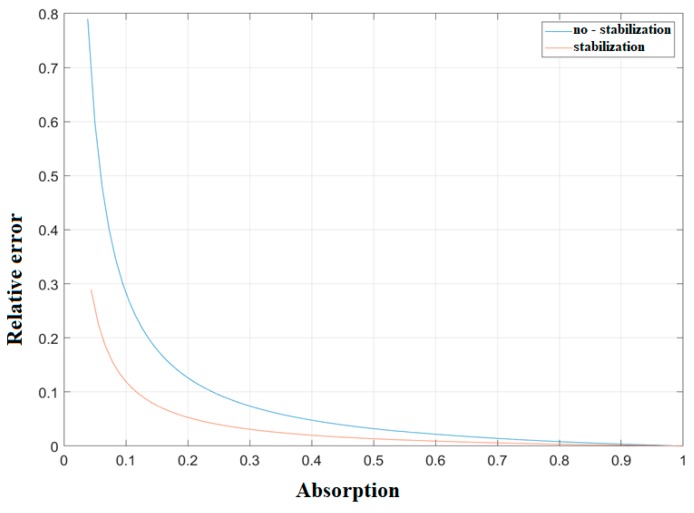
Simulated relative error vs the sound absorption value.

Figure 7 shows a well-defined relationship between the measured absorption value and the error related to it, which is certainly influenced in absolute terms by the displacements with respect to the equilibrium.

## 5. Discussions and Conclusions

The present paper reported a new measurement system for the acoustic absorption of a pavement developed inside the LIFE15 ENV/IT/000268 NEREiDE project. The new method is derived from the Adrienne one (ISO 13472-1), but the sensor used is a *p*-*u* probe mounted on a vehicle moving over the road surface. With this important changes, it is now possible to measure acoustic absorption coefficient on-site, in a contactless way along all the site. Furthermore, it has been shown that the resulting frequency bandwidth is wider.

The correct set-up of the instrumentation has been studied, with particular emphasis on the height of the instrumentation above the ground. It has been shown that the ideal height, at which the lowest measurement error is achieved, is 0.16 m, slightly different from the 0.25 m used in the Adrienne method.

The present paper mainly reported the solution applied to overcome the greater difficulty for this kind of measurements, i.e., the stabilization and damping of the system, required in a vehicle in motion. The instrumentation has been uncoupled from the vehicle’s frame with a system studied to maintain constant the distance from the ground. As reported, a PID (Proportional Integral Derivative) controller was used to act on the error given by the difference of the target position and the real position of the measurement system with respect to the pavement. The test performed showed good results for keeping the instrumentation at a fixed height within an order of magnitude of 1 cm, which should be enough to achieve good accuracy in the evaluation of the absorption coefficient. For completeness, the extent of the influence is currently under study. The laboratory tests showed an excellent behavior of the stabilizer in dynamic conditions, perfectly within what was initially expected. Field measurements necessarily suffer from a greater uncertainty, which however allows to the use of the system within good margins for acoustic characterizations. In particular, the implemented possibility of obtaining the distance data from the system itself is rather useful, although it has not yet been tested. In the future, the authors expect to use the data for a possible real time correction to the calculations of the parameters that may depend on the distance.

This study improves on the work of Tijs and de Bree [53] by introducing a stabilization, but also allows the measurements at lower frequency by using a set-up similar to the Adrienne method. The required higher distance from probe to surface is safer for the instrumentation but also indirectly leads to a greater surface under study. However, this set-up implies a bigger source-pavement distance that allows a better approximation of the loudspeaker as point source on both the high and low frequencies. In addition, the methodology presented can be also considered as an improvement of the Adrienne method itself because it goes beyond the need to subtract the signals over time, resulting mathematically easier to operate and analyze.

At present, the system is designed to work at low speeds, within 30 km/h. Placed in the wake of the vehicle, the effect of the wind on the sensor is negligible, also because the sensor is equipped with a special windproof cap that gives it additional protection. In addition, the sensor is located inside an amplification horn, with the axis perpendicular to the ground, where a small windproof layer placed on the entrance and exit significantly reduces the disturbing effects generated by wind turbulence. In addition to this, the source power is sufficient to make other noise sources per se negligible. In particular, the speed sensor is highly directional, and its direction of measure is perpendicular to the road surface, thus further reducing the rolling noise produced by the wheel. Specific laboratory tests, not reported in this article, have shown effective independence of the probe measurement from sources placed outside the axis and of power comparable to the tire/road emission at the speeds investigated. While the calibration of a microphone can be performed with a suitable calibrator, no standard speed references to rely on exists for speed probes. For this purpose, the authors’ actual efforts and future developments will investigate the development of a calibration system. This phase needs to measure the acoustic impedance, which is given by the ratio between the velocity values of the particles and pressure measured at the same point. The calibration measurement will be performed inside an anechoic chamber or inside a stationary wave tube (Kundt tube) [63]. Another method could be to perform a comparative measure with the Adrienne method, estimating the absorption coefficient of a material from its surface acoustic impedance.

Finally, the authors do not exclude the study of the new presented methodology also using a suitable *p*-*p* sensor, which can still be a reliable, fast, and convenient tool for on-site measurements.

## Figures and Tables

**Table 1:** Comparison of in-situ measurement methods.

	Adrienne Method	Adopted Method Based On *P*-*U* Probe
**In-Situ Measurement**	✓	✓
**Contactless Measurement**	✓	✓
**Frequency Bandwidth**	250 Hz ÷ 4 kHz	315 Hz ÷ 10 kHz
**Exposed Area Diameter**	≈ 1.4 m	≈ 1.4 m
**Height of The Sound Source**	1.25 m	1.5 m
**Height of The Sound Microphone / *P*-*U* Probe**	0.25 m	0.16 m
**Absorption Coefficient Sensitivity to Receiver Height Variation (F ≥ 315)**	2.4 m^−1^	2.1 m^−1^
**Absorption Coefficient Sensitivity to Source Height Variation (F ≥ 315)**	0.5 m^−1^	0.3 m^−1^

**Table 2:** RMS (Root mean square) of the average displacement measured at 30 km/h.

	RMS of Average Displacement [mm]
No stabilization	11.5
Stabilization	4.7
